# ZNF521 Enhances MLL-AF9-Dependent Hematopoietic Stem Cell Transformation in Acute Myeloid Leukemias by Altering the Gene Expression Landscape †

**DOI:** 10.3390/ijms221910814

**Published:** 2021-10-06

**Authors:** Emanuela Chiarella, Annamaria Aloisio, Stefania Scicchitano, Katia Todoerti, Emanuela G. Cosentino, Daniela Lico, Antonino Neri, Nicola Amodio, Heather Mandy Bond, Maria Mesuraca

**Affiliations:** 1 Department of Experimental and Clinical Medicine, University Magna Græcia, 88100 Catanzaro, Italy; aloisio@unicz.it (A.A.); scicchitano@unicz.it (S.S.); emanuelagiuliana.cosentino@studenti.unicz.it (E.G.C.); amodio@unicz.it (N.A.); 2 Hematology, Fondazione IRCCS Ca’ Granda Ospedale Maggiore Policlinico, 20122 Milan, Italy; katiatodoerti@gmail.com (K.T.); antonino.neri@unimi.it (A.N.); 3 Department of Oncology and Hemato-Oncology, University of Milan, 20122 Milan, Italy; 4 Exiris S.r.l., 00128 Roma, Italy; 5 Department of Hematology, Cancer Research Centre Groningen, University Medical Centre Groningen, University of Groningen, 9712 CP Groningen, The Netherlands; 6 Department of Obstetrics and Gynaecology, Pugliese-Ciaccio Hospital, University Magna Græcia, 88100 Catanzaro, Italy; Dani.li@hotmail.it

**Keywords:** acute myeloid leukemia (AML), cord blood-derived hematopoietic stem cells (CB-CD34^+^), human zinc finger protein 521 (hZNF521), mixed lineage leukemia gene (MLL) AF9 (MLLT3 or LTG9), fusion gene MLL-AF9, chromosomal translocations, gene expression

## Abstract

Leukemias derived from the MLL-AF9 rearrangement rely on dysfunctional transcriptional networks. ZNF521, a transcription co-factor implicated in the control of hematopoiesis, has been proposed to sustain leukemic transformation in collaboration with other oncogenes. Here, we demonstrate that ZNF521 mRNA levels correlate with specific genetic aberrations: in particular, the highest expression is observed in AMLs bearing MLL rearrangements, while the lowest is detected in AMLs with FLT3-ITD, NPM1, or CEBPα double mutations. In cord blood-derived CD34^+^ cells, enforced expression of ZNF521 provides a significant proliferative advantage and enhances MLL-AF9 effects on the induction of proliferation and the expansion of leukemic progenitor cells. Transcriptome analysis of primary CD34^+^ cultures displayed subsets of genes up-regulated by MLL-AF9 or ZNF521 single transgene overexpression as well as in MLL-AF9/ZNF521 combinations, at either the early or late time points of an in vitro leukemogenesis model. The silencing of ZNF521 in the MLL-AF9 + THP-1 cell line coherently results in an impairment of growth and clonogenicity, recapitulating the effects observed in primary cells. Taken together, these results underscore a role for ZNF521 in sustaining the self-renewal of the immature AML compartment, most likely through the perturbation of the gene expression landscape, which ultimately favors the expansion of MLL-AF9-transformed leukemic clones.

## 1. Introduction

Acute myeloid leukemias (AMLs) are characterized by chromosomal translocations involving the Mixed Lineage Leukemia (MLL) gene, which results in a variety of fusion oncogenes [1] derived from genes that are normally required during hematopoietic development; once fused, they induce epigenetic and transcription factor dysregulation [2]. One of the most frequent MLL translocations leading to AML results from the chromosomal translocation of t(9;11) (p21–22;q23), which fuses the MLL gene with the AF9 gene (also known as MLLT3 or LTG9) and is considered an initiating factor for leukemogenesis [3,4,5]. The tumorigenic potential of MLL-AF9 fusion mainly results in a monocytic immune phenotype that has an aggressive course with frequent relapses and a short survival time [6,7].

The MLL-AF9 fusion gene is described as playing a critical role in stem and progenitor populations and in leukemogenesis [8,9,10,11,12]. During hematopoietic cell development, MLL and AF9 wild-type proteins act as components of protein complexes leading to the transcriptional initiation (MLL) and elongation (AF9) of target genes through the methylation of specific histones, resulting in activated promoters [13,14]. MLL-AF9 target genes include transcription factors from the Hox family (HoxA9, HoxA10, HoxA5, ZNF532, HoxA7, Meis1 and PBX3) [15,16,17,18]. This set of target genes was also confirmed in leukemias induced in mice transformed by MLL-AF9 (HoxA9, HoxA10, Evi1 (MECOM), HoxA2, HoxA7 HoxA3, and HoxA13) and also induced iMLL-AF9 in mice HoxA9, HoxA10, Meis1, Eya1, Mef2c, Myb, and Six1 [7,19]. In an MLL-AF9 transcriptionally inducible mouse model, HoxA9, Meis1, Mef2c, Cdk4, Cdk6, Spi1, and Cebpβ were identified as being significantly dysregulated [20]. This multitude of evidence has established that the activation of the Hox family, together with the β-catenin signalling pathway [13,21], is required for leukemogenic transformation depending on MLL-AF9.

Concerning the role of ZNF521, several gene expression profiling data sets have revealed high expression, especially in leukemic patients with MLL rearrangements compared to other leukemias [22,23]. The silencing of MLL-AF9 in the THP-1 AML cell line leads to a coherent down-regulation of ZNF521 [24]. Epigenetic analyses in THP-1 cells identified both the Hox locus and the ZNF521 promoter as having methylated H3K79me2 histones resulting from the MLL-AF9 fusion oncogene [25]; moreover, ChIp experiments detected MLL-AF9 binding sites in the promoter of HOX family members as well as in the ZNF521, ZNF433, and ZNF532 transcription factors [14]. Interestingly, a thorough analysis of the ZNF521 promoter highlighted a critical region of 1689bp that is activated by MLL-AF9 and MLL-ENL, suggesting a positive forward loop [26]; moreover, a comparative transcriptomic expression approach implicated hZNF521/mZfp521 as a conserved Hematopoietic Stem Cell (HSC)-enriched transcription factor in human and murine hematopoiesis compared to in downstream progenitor and effector cells [27]. Of note, the transcription factors HLF, MECOM, MEIS1, PRDM16, HOXA9 as well as ZNF521 were identified as MLL-AF9 targets that are highly expressed in patients with MLL rearrangements [27].

ZNF521 is known to be highly expressed in the immature CD34^+^ compartment of hematopoietic cells [28]. An extensive eQTL-based analysis of the candidate regulators of hematopoiesis identified mZfp521 in the top-regulator locus of the hematopoietic pool [29]. Competitive serial transplantation assays in KO ZFP521^−/−^ mouse fetal liver cells showed that mZfp521 increases HSC frequency and causes a significant reduction in both common myeloid and granulocyte-macrophage progenitors; when, these mZFP521-deficient mice where transduced with MLL-AF9, they displayed a delay in leukemogenesis [27].

In normal hematopoiesis, hZNF521 mRNA rapidly declines when CD34^+^ is induced to differentiate it and becomes weak or undetectable when these progenitors progress towards erythroid [30], granulocytic [31], or B-lymphoid differentiation [32,33]. Conversely, in leukemic-transformed cells, the expression of ZNF521 is maintained and is significantly high in many AML cases with MLL gene rearrangements [26,28,34].

In this manuscript, AML gene expression profile datasets were interrogated for hZNF521 expression, and its correlation with specific gene rearrangements and mutations was also addressed. In vitro, we found that the enforced co-expression of ZNF521 and the MLL-AF9 fusion oncogene results in an increased effect on the proliferation of CB-CD34^+^ cells that is likely derived from an increased progenitor cell population. Transcriptome profiling of CD34^+^ cells transduced with either MLL-AF9, ZNF521, or a combination of the two transgenes highlighted specific sets of up- or down-regulated genes that are involved in the leukemic phenotype, likely implying a cooperative effect for these two transcription factors.

## 2. Results

### 2.1. Differential Expression of the Transcription Co-Factor ZNF521 in AMLs: Correlation with Specific Cytogenetic Profiles

**Figure 1:**
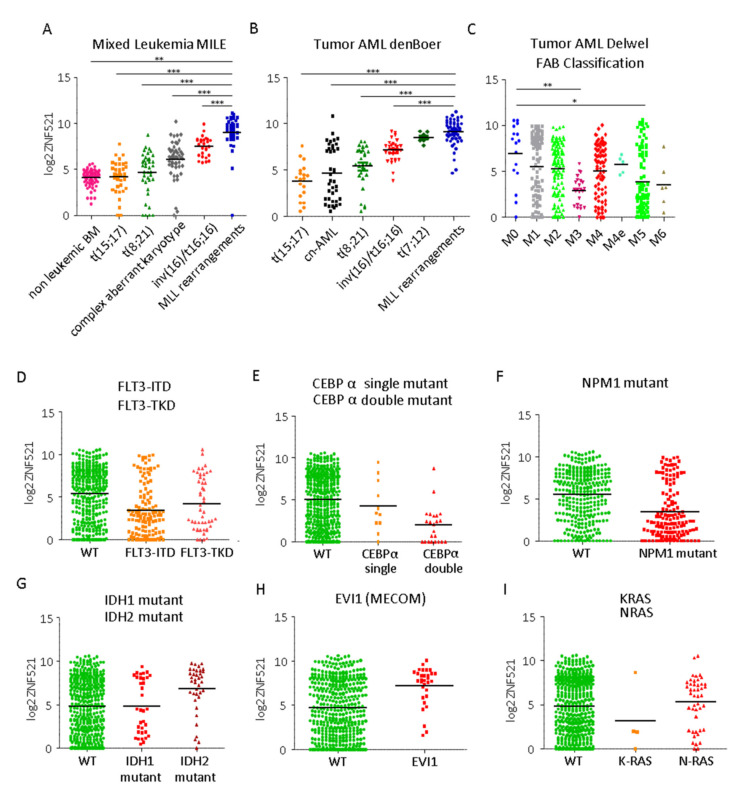
ZNF521 gene expression levels according to cytogenetic groups and FAB classification. (**A**) ZNF521 mRNA expression levels were analyzed in different cytogenetic groups compared to normal karyotype AMLs from the MILE data set (GSE13204). Significant *p* values: MLL rearrangement vs. non leukemic bone marrow, *p* = 8.85 × 10^–3^; MLL vs. t(15;17), *p* = 3.97 × 10^–4^; MLL vs. t(8;21), *p* = 7.01 × 10^–4^; MLL vs. complex aberrant karyotype, *p* = 2.381 × 10^–9^; and MLL vs. inv(16)/t(16;16), *p* = 2.611 × 10^–3^. (**B**) den Boer data set (GSE17855): Significant *p* values: MLL vs. t(15;17), *p* = 1.15 × 10^–18^; MLL vs. cn–AML, *p* = 1.31 × 10^–14^; MLL vs. t(8;21), *p* = 3.73 × 10^–13^; 16)/t(16;16), *p* = 5.03 × 10^–8^. (**C**) ZNF521 mRNA expression analyzed with respect to FAB subgroup classification of AMLs (GSE6891). Significant *p* values: MO vs. M3, *p* = 6.02 × 10^–3^ and MO vs. M5 *p*= 4.47 × 10^–2^. ZNF521 mRNA expression level in 460 pediatric and adult AML samples (Delwel) stratified with respect to molecular lesions: (**D**) FLT3, (**E**) CEBPα, (**F**) NPM1, (**G**) IDH1 mutations, (**H**) EVI1 (MECOM) aberrant expression and (**I**) RAS oncogenes. (cn: cytogenetically normal). Significant *p* values: * < 0.05, ** < 0.01, *** < 0.001.

In order to better define the role of ZNF521 in AML, we examined ZNF521 mRNA expression levels in relation to some clinical/pathological covariates using publicly available datasets. The highest ZNF521 mRNA expression levels were observed in AMLs involving t(11q23) MLL rearrangements in two distinct datasets (MILE and den Boer) [35,36] (Figure 1A,B); elevated ZNF521 mRNA expression levels were also evidenced in AMLs with t(7;12) or that were carrying internal rearrangements of chromosome 16.

Conversely, relatively low ZNF521 expression levels appeared to correlate with the t(8;21) translocation associated with the AML1-ETO fusion gene or the t(15;17) translocation generating the PML-RARα genes (Figure 1A,B).

When considering the FAB subgroups, the highest levels of ZNF521 mRNA expression were observed in M0, while the lowest amounts were expressed in M3 AMLs (*p* = 6.02 × 10^−3^), indicating that elevated ZNF521 expression correlates with minimally differentiated acute myeloblastic leukemia (M0-AML), a rare type of AML that is associated with poor prognosis (GSE6891) (Figure 1C).

Additionally, ZNF521 expression levels were investigated in relation to specific gene mutations and aberrations commonly found in AMLs [37]: interestingly, lower expression was observed in association with internal tandem duplications (ITD) in the Fms-related receptor tyrosine kinase 3 (FLT3) gene (*p*-value = 2.49 × 10^−9^) rather than with the presence of mutations in the activation loop of the tyrosine kinase domain (FLT3-TKD) (*p*-value = 0.129) or with the CCAAT/enhancer-binding protein alpha (CEBPα) gene (double (*p*-value = 2.54 × 10^−6^) or single (*p*-value = 0.658) mutations; nucleophosmin (NPM1) gene mutations were associated with a lower ZNF521 level in comparison to the normal wild-type (WT) condition (Figure 1D–F). In contrast, higher expression levels were associated with the presence of mutations in the isocitrate dehydrogenase 2 (IDH2) gene but not in IDH1 (Figure 1G). The aberrant expression of ecotropic virus integration site 1 (EVI1 MECOM) (Figure 1H) was associated with a higher expression of ZNF521. Notably, EVI1 correlates to MLL rearrangements in AMLs [38] and with the Hox locus in particular [7,16,38,39,40], which has been identified as a MA9 target.

Finally, similar ZNF521 mRNA expression levels were observed in AMLs bearing mutated RAS (KRAS or NRAS) compared to the WT counterpart (Figure 1I).

### 2.2. Over-Expression of Both MLL-AF9 and ZNF521 Genes Increases the Expansion of Umbilical CB-Derived Early Hematopoietic Progenitors In Vitro

**Figure 2:**
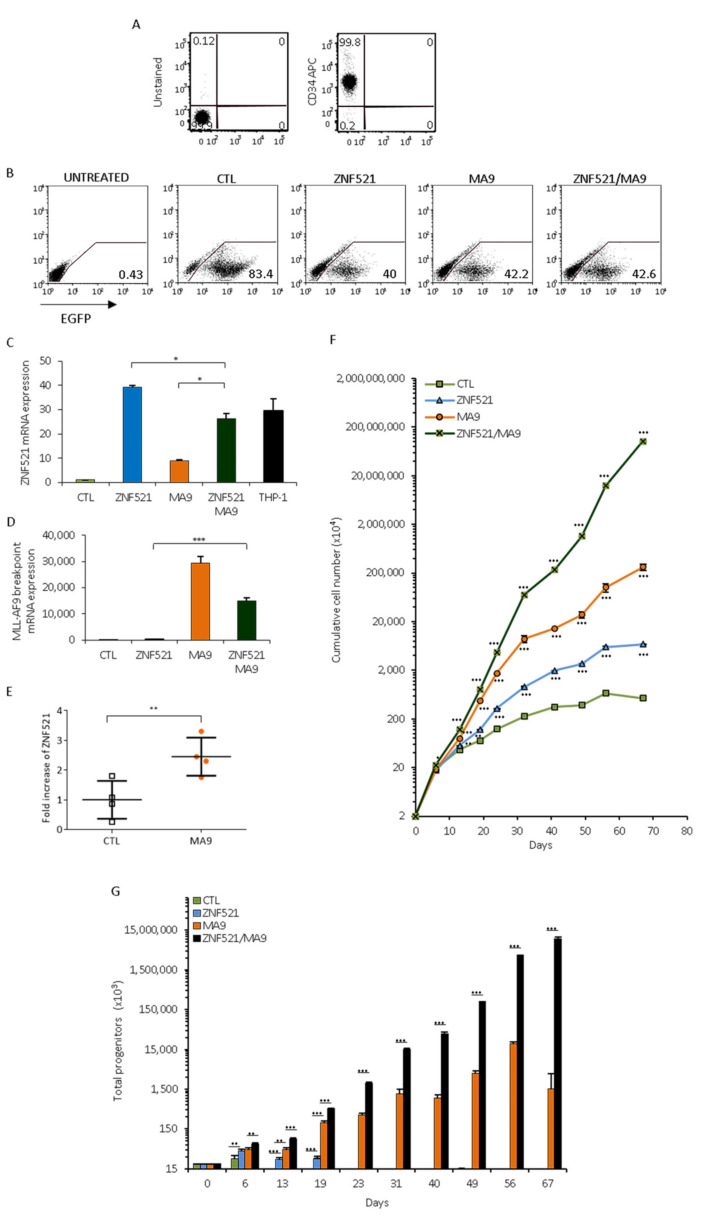
CB-CD34^+^ transduced cell expansion in vitro.(**A**) Purified cord blood hematopoietic progenitor cells were characterized by FACS for the CD34 antigen. (**B**) FACS analysis of transduced cells was performed to evaluate the percentage of E-GFP positive cells at 6 days after transduction. The transduction efficiency of CD34^+^ cells was evaluated by Q-RT-PCR analysis: mRNA levels of (**C**) ZNF521 and (**D**) the MLL-fusion gene are significantly enhanced in transduced cells compared to control cells. (**E**) In four different MLL-AF9 transductions of CB-CD34^+^ cells, the amounts of ZNF521 mRNA are shown (analyzed at 37 days). (**F**) CB-CD34^+^ cells (2 × 10^4^) were plated in MyeloCult™ complete with cytokines. Cumulative cell expansion is shown, and the *p*-value is calculated compared to control vector. All assays were performed in triplicate. (**G**) Colony-forming cell (CFC) assays were performed using StemMACS HSC-CFU medium complete with cytokines. Colonies were scored after 2 weeks, and the number of progenitors was normalized to the total cell number in the relevant culture at the time of plating. Data are represented as mean ± SD (* *p* < 0.05, ** *p* < 0.01, *** *p* < 0.001).

To investigate the potential role of ZNF521 in the regulation of leukemic stem cells with MLL rearrangements, we transduced hematopoietic progenitor hCB-CD34^+^ cells (Figure 2A) with a lentivirus expressing the MLL-AF9 fusion gene (UMG-LV6-MA9) alone or in combination with ZNF521 (UMG-LV6-ZNF521), in comparison to cells transduced by UMG-LV6 expressing only E-GFP. Six days after infection, the transduction efficiency was verified by E-GFP expression, which was determined by means of a FACS analysis resulting in about 50% positive cells (Figure 2B). Q-RT-PCR analysis confirmed that ZNF521 transcript levels were increased in cells infected with UMG-LV6-ZNF521 and in CD34^+^ cells co-transduced with UMG-LV6-MA9 and UMG-LV6-ZNF521. The endogenous levels of ZNF521 were enhanced in the cells overexpressing the fusion protein MA9 (Figure 2C). Similarly, the MLL-AF9 transcript was overexpressed and detected by the primers the span the breakpoint of the translocation in the populations transduced either with the lentiviral vector UMG-LV6-MA9 alone or together with UMG-LV6-ZNF521 (Figure 2D).

After transduction, the cells were plated for proliferation assay and underwent phenotypic analysis: when the MLL-AF9 fusion gene was introduced, an increase in the ZNF521 endogenous transcript (2.5×) in CB-CD34^+^ cells was detected by Q-RT-PCR compared to the control cells after 37 days of culture (Figure 2E).

During cell culture expansion, the number of ZNF521-overexpressing cells significantly increased compared to the control; however, these cells did not immortalize and had a limited life span, and after 56 days, they ceased to grow (Figure 2F). Instead, enforced expression of the MLL-AF9 fusion protein was able to immortalize the human CB-CD34^+^ cells, conferring a strong proliferative advantage; in particular, MLL-AF9 expressing cells steadily increased in number beginning from the second week. Something noteworthy is that double transduction with ZNF521 and MLL-AF9 transgenes led to a further increase in the cumulative number of cells (382× more than MA9 alone); the transformed cell populations reproducibly continued to grow after 2 months in liquid cultures (Figure 2F).

Colony assays were performed at specific intervals during culture expansion (Figure 2G), allowing the total number of progenitors to be calculated. Enforced expression of ZNF521 alone produced a limited increase in progenitors (twofold), as evidenced from the colonies derived from the first 3-week period alone; importantly, when the CD34^+^ cells were transduced with MA9, the number of hematopoietic progenitors was potently enhanced due to the continued expansion of transformed hematopoietic stem cells derived from the colonies formed at different intervals during the culture expansion. This effect was even more potent when the CD34^+^ cells were transduced with the combination of the two factors MLL-AF9 and ZNF521 (Figure 2G). Taken together, these data demonstrate that MLL-AF9/ZNF521 co-expression produces an enhancement in early hematopoietic progenitors compared to each single transgene, which consistently gave rise to an increased number of colonies, suggesting that ZNF521/MLL-AF9 combination is crucial to promote the expansion of the transformed progenitor cell population during leukemogenesis (Figure 2G).

### 2.3. Transcriptome Profiling of ZNF521, MA9 and Double ZNF521/MA9 Transductions

The global transcript expression profiles of ZNF521 or MA9 overexpressing cells were investigated in comparison to control cells at 23 days, whereas ZNF521-MA9 double transduced cells were analyzed both at day 23 and 58 of our in vitro leukemogenesis model.

**Figure 3:**
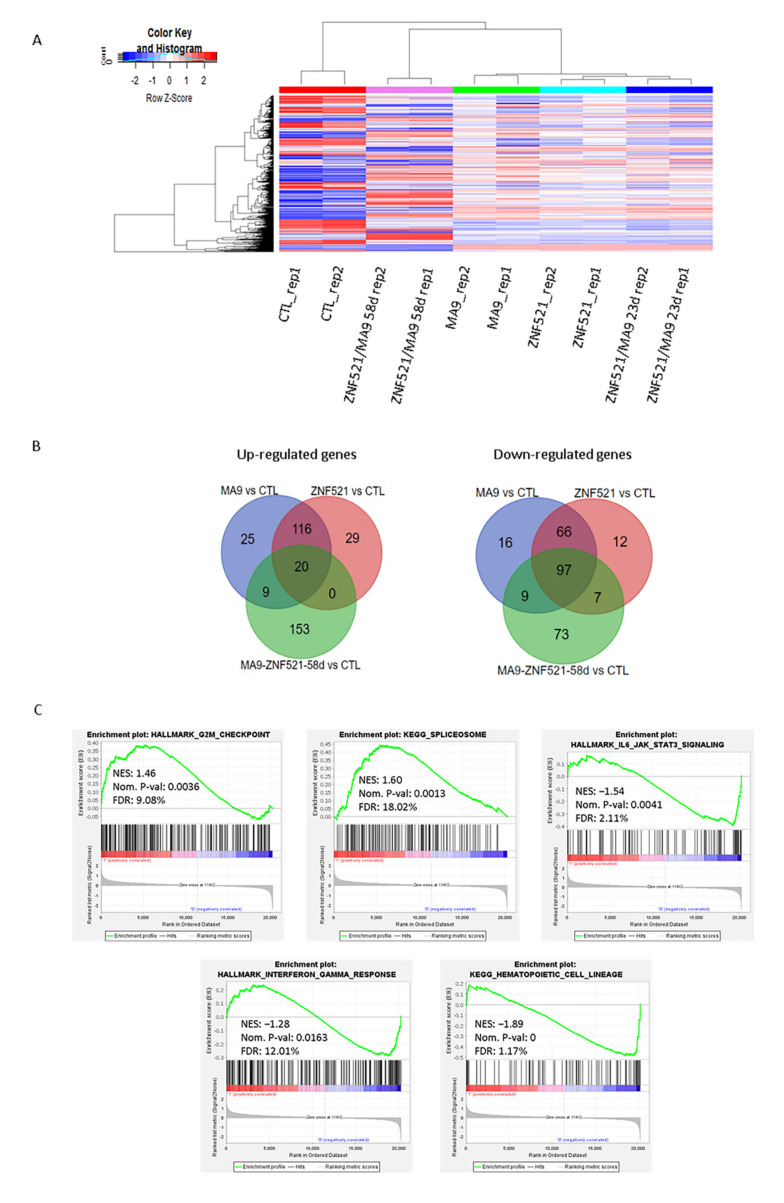
Gene expression analysis was performed with the ClariomD array. (**A**) Clustering on the top 1% (1358/135750) of transcripts with the highest variance across the entire dataset. Average clustering method was applied. Blue–red color scale was used to set rows with a mean of zero and a standard deviation of one. (**B**) Venn diagrams illustrating the significantly up- or down-regulated genes at FDR <10% by RankProduct analysis in ZNF521 and MA9 at 23 days or after their combination at 58 days compared to control cells. The corresponding transcript lists are reported in Supplementary File S2. (**C**) Selected significant Hallmark and Kegg pathways determined by GSEA analysis with corresponding normalized enrichment score (NES) and *p*-value parameters.

The hierarchical clustering analysis (Figure 3A) for the top 1% of transcripts at the highest variance across all samples revealed a more similar global expression pattern in the single- or double-transduced cells at 23 days with respect to ZNF521-MA9 at 58 days compared to the control samples at 23 days (Supplementary File S1 and Supplementary Table S1). Additionally, supervised analysis revealed differentially expressed (DE) transcripts that significantly distinguished each treated group in comparison to the control condition. The corresponding lists that were obtained at a percentage of false positive (pfp) that was less than <10% by RankProduct analysis are shown in Supplementary File S1. Moreover, aiming to define those transcripts specifically modulated in ZNF521-MA9 combination at 23 days and 58 days or that were commonly found either in ZNF521 or MA9 alone at 23 days compared to the control condition, the transcript lists resulting from these three comparisons were matched (Supplementary File S2 (1,2)). In detail, we found 20 genes that were up-regulated in all three comparisons, 29 genes that were specifically up-regulated by ZNF521, and 25 genes that were up-regulated by MA9, whereas a wider list of 153 transcripts were found to be positively modulated in the ZNF521-MA9 combination at 58 days, as shown by Venn diagrams [41]. Moreover, a higher number of genes were found to be commonly negatively modulated in all conditions (97 genes), with few genes being specifically modulated in each single condition (16 genes in MA9, 12 genes in ZNF521), while 73 genes were specifically down-regulated in ZNF521-MA9 at 58 days (Figure 3B and Supplementary File S2 (2)).

In order to find which functional categories were significantly modulated by ZNF521 in the context of MA9, we considered the transcripts that resulted as being differentially expressed in both MA9- and ZNF521-transduced cells compared to in the control condition.

In particular, we analyzed 136 positively and 163 negatively modulated transcripts resulting from both the MA9 and ZNF521 comparisons using DAVID 6.8 functional annotation clustering analysis.

Notably, nine annotation clusters of up-regulated genes were significantly enriched in the GO-terms associated with hematopoietic development and erythroid cell lineage differentiation together with cell–cell adhesion (Supplementary Table S2, up).

Furthermore, 31 significant annotation clusters were identified for commonly down-regulated transcripts, resulting from enriched GO-terms related to immune response regulation, cytokine metabolism and secretion, peptidase activity, and sterol metabolism and storage together with biological processes concerning lymphocyte proliferation and cell death (Supplementary Table S2, below). Finally, the biological processes of cell migration and genes codifying for extracellular exosome cellular components were shown to be globally enriched by both analyses (Supplementary Table S2, below).

In order to find the molecular pathways that are specifically modulated by the ZNF251-MA9 combination at the later time points and that potentially contribute to a full leukemic phenotype, the global transcript expression patterns of transduced compared to empty vector controls were analyzed by means of gene set enrichment analysis (GSEA) (Figure 3C).

In detail, several gene sets associated with cell cycle, protein, and RNA metabolism were found to be positively modulated, whereas groups of genes involved in various cytokine signaling or cellular metabolic pathways were found to be down-regulated under the ZNF521-MA9 combination at 58 days in comparison to the control conditions (Supplementary File S3).

Functional annotation clustering analysis was performed using the DAVID 6.8 tool [42] on 226 transcripts that were found to be specifically modulated at the later time point in ZNF251-MA9 compared to the controls. In particular, 28 annotation clusters of GO-terms were significantly enriched at high stringent conditions and involved several biological processes, such as the positive regulation of cytokine production and signal transduction, together with the genes implicated in the regulation of locomotion and leucocyte cell–cell adhesion and activation (Supplementary File S4).

### 2.4. Transcript Analysis for MLL-AF9 Targets

**Figure 4:**
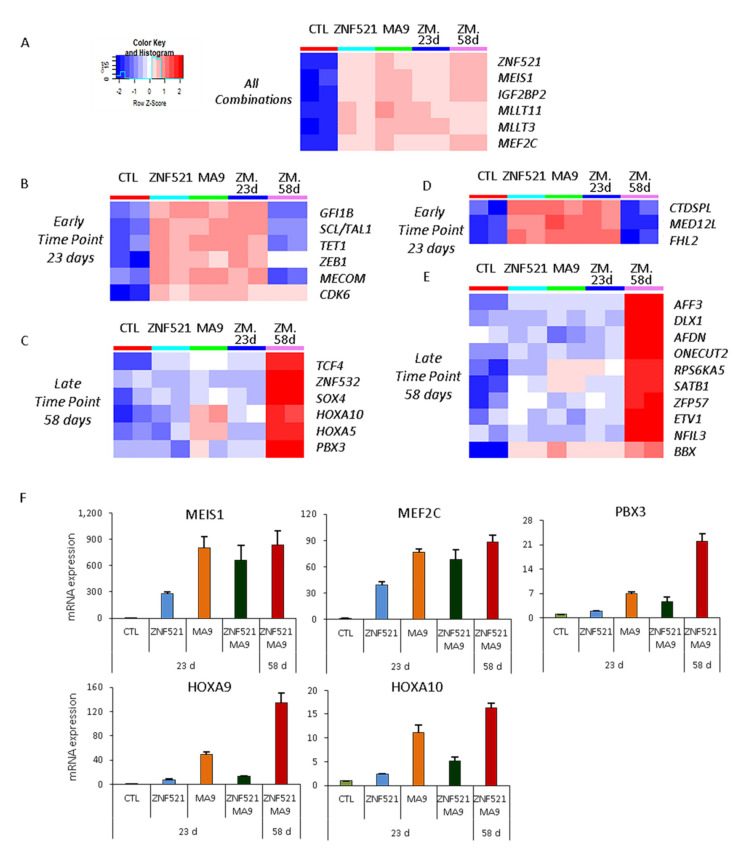
Heatmaps of selected transcripts from differentially expressed transcript lists at FDR < 10% of ZNF521, MA9, and ZNF521-MA9 (ZM) at 23 and 58 days compared to the control condition at 23 days. Known or other target genes that are up-regulated in all comparisons (**A**) at early (**B**,**D**) or late time points (**C**,**E**) are depicted. Blue–red color scale was used to set rows with a mean of zero and a standard deviation of one. (**F**) Confirmation of the array by Q-RT-PCR for MEIS1, MEF2C, HOXA10, HOXA9, and PBX3.

The global transcript expression levels in differentiating cells transduced by ZNF521, MA9, and ZNF521-MA9 (ZM) compared to the control condition were defined by gene expression profiling by means of a high-density ClariomD array (Figure 4A–E). Particularly, we found the up-regulation of known MA9 target genes, such as MEIS1, IGF2BP2, MLLT11, MLLT3, and MEF2C, including ZNF521, in all combinations (Figure 4A) [14,23,24,25,26,27]. The double transduction at 58 days showed the largest number of transcripts that were distinctively up-regulated in comparison to the control condition. Among the 153 specific up-regulated genes, we found several MA9 target genes, such as HOXA5, HOXA10, and PBX3 [14,15] (Figure 4C, Supplementary Table S1) together with the TCF4, ZNF532, and SOX4 transcription factors. The genes, ZNF521, MEIS1, IGF2BP2, MLLT11, MLLT3, and MEF2C, were found (Figure 4A, Table S1) to be induced in all conditions, especially by MA9 alone, supporting the occurrence of a reciprocal feedback transcriptional loop between ZNF521 and MA9 (Supplementary Table S1).

MA9 targets are frequently Hox family genes, and accordingly, the genes for HOXA5, HOXA10, and PBX3 were induced by MA9 alone and had higher levels 58 days after being initially detected under less stringent conditions (FDR > 10%) by microarray analysis. The MA9 targets MEIS1, MEF2C, and PBX3 and the HOXA9/HOXA10 genes were validated by Q-RT-PCR, confirming the microarray data showing that MA9 and ZNF521 already up-regulates MEIS1 and MEF2C at day 23; instead, the HOXA9/HOXA10 and PBX3 genes are up-regulated by MA9 but not by ZNF521 (Figure 4F).

In addition to the core MLL-AF9 targets, IGF2BP2, also reported by Horton et al. [23], was found up-regulated in all combinations (Figure 4, Supplementary Table S1). Notably, IGF2BP2 has been shown to support the survival and cycling of hematopoietic stem cells and acts as an oncogene in various tumors [43,44].

Heatmap analysis illustrates that there are groups of genes up-regulated at the early time point (23 days) and that the others are up-regulated at the later time point (58 days) (Figure 4B–E). The genes that had either been up-regulated at day 23 or that could already be described as MA9 targets include GFI1B, SCL/TAL, TET1, ZEB1, and MECOM. The genes that appeared later, by day 58, TCF4, ZNF532, SOX4, HOXA10, HOXA5, and PBX3, could also be classified as MA9 targets (Figure 4B,C and Supplementary Table S1).

New MA9 targets resulting among early modulated transcripts were CTDSPL, MED12L, and FHL2 (Figure 4D and Supplementary File S3), and up-regulaAFF3, DLX1, AFDN, RPS6KA5, ONECUT2, SATB1, ZFP57, ETV1, NFIL3, and BBX were shown to be more strongly up-regulated at a later timepoint (Figure 4E and Supplementary File S3).

Among the genes up-regulated at the early time point by both ZNF521 or MA9, we found: (i) GFI1b, which acts as a transcriptional repressor in the hematopoietic lineage for GATA-1, Runx1, and histone deacetylase (HDAC); (ii) TAL1, which acts as a regulator of hematopoiesis and whose targets include c-Kit and UTX demethylase [45]; (iii) the TET1 gene, an oncoprotein in AML, which acts in a STAT1/TET1 axis [26,46].

ZNF521 overexpression seems to correlate with an increase of ZEB1 expression, an epithelial–mesenchymal transition (EMT) transcription factor (Figure 4B and Supplementary Table S1). ZEB1 can act together with Snail and Twist to induce EMT in solid tumors [47,48]. These transcription co-factors interact with the nucleosome remodeling and deacetylation (NuRD) complex [47,49,50]. We have shown that ZNF521 interacts with the NuRD complex for transcriptional repression [30,51]. Recently, there have been two manuscripts delineating the role for ZEB1 in HSCs and their differentiation using models with Zeb1^−/−^, where there was an acceleration of MLL-AF9 and Meis1a/Hoxa9-driven AML progression, implicating Zeb1 as a tumor suppressor in AML LSCs [52]. In AML with a high expression of ZEB1, AML is closely correlated with poor patient prognosis, and mechanistically, it was found that ZEB1 can interact with P53 and can regulate the PTEN/PI3K/AKT signaling pathway [53].

MECOM/EVI1, CDK6, and TCF4 have been described as specific MLL-AF9 targets and are up-regulated here by day 23 (MECOM/EVI1, CDK6), whereas TCF4 is detected later, at day 58. TCF4 is a DNA-binding transcription factor that is targeted by RUNX1 and MLL-AF9 and that is considered an independent prognostic factor in AML, while CDK6 is involved in the cell cycle.

To delineate the effect of ZNF521 and MA9 in this model, we also focused on the genes known to promote and maintain the leukemic phenotype in MLL-AF9 leukemias, including those encoding transcription factors, epigenetic modulators, and cell cycle regulators as well as those involved in the transport or uptake of nutrients, which are summarized in Supplementary Table S1. Many of these genes are associated with poor prognosis in AML patients and have hazard values above 1 (Gepia) [54].

In addition to the transcription factors already noted to be induced by MLL-AF9, there are a set of up-regulated transcriptional factors (Supplementary Table S1 (1,2)), which are often known to play roles in self-renewal, differentiation systems, and in other cancers. The induction of transcriptional activation can indirectly result in a multi-factorial cascade effect that is required for leukemic transformation.

**Figure 5:**
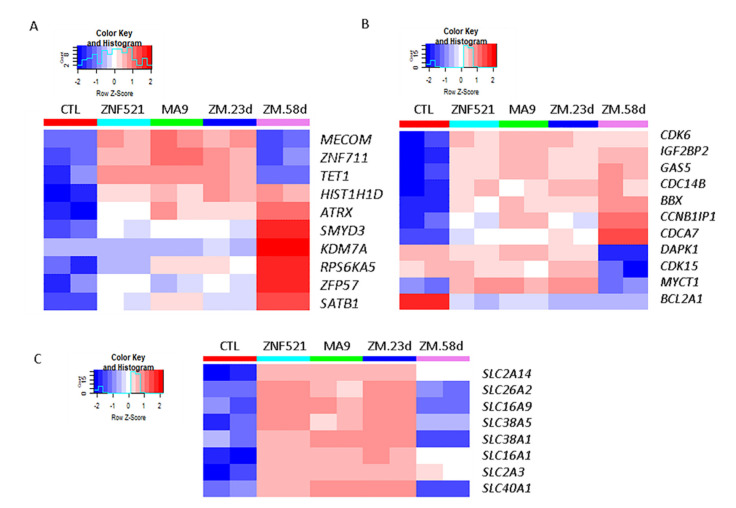
Heatmaps of selected differentially expressed transcripts in ZNF521, MA9, and ZNF521-MA9 (ZM) at 23 days and/or in ZNF521-MA9 (ZM) at 58 days compared to the control condition. (**A**) Genes involved in epigenetic functions. (**B**) Genes involved in cell cycle function. (**C**) Transporter genes. Blue–red color scale was used to set rows with a mean of zero and a standard deviation of one.

MLL-AF9 relies on the epigenetic activity of AF9 to modulate histone methylation and to recruit DOT1L to target genes in HSCs and to sustain levels of H3K79me2 [13]. Here, additional genes are found up-regulated, performing a variety of epigenetic activities: histone demethylase, methyltransferase, methylcytosine dioxygenase 1, ATPase/helicase, and the recruitment of factors for chromatin-remodeling, which together, are likely to collaborate with the MA9 epigenetic genome transformation (Figure 5A and Supplementary Table S1 (3)).

Considering the increase in proliferation upon ZNF521-MA9 combination, we also focused on genes associated with the cell cycle and apoptosis. In addition to the up-regulation of the MA9 target, Cdk6 [14,20,26], there was an increase of six genes known to promote the cell cycle and down-regulation of four genes for apoptosis (Figure 5B and Supplementary Table S1 (4)). The GSEA analysis performed on the genes that were differentially expressed between the ZNF521-MA9 combination and ZNF521 is reported in Supplementary File S5A. Several significantly up-regulated GO gene sets in MA9-ZNF521 at 58 days compared to ZNF521-traduced cells at 23 days were associated with the regulation of cell growth and thus were possibly associated with the modulation of the clonogenic capacity in double- compared to single-transduced cells (Supplementary File S5B–C).

Highly proliferating leukemic cells require an increase uptake of nutrients for high glycolytic and glutamine metabolism. There was an up-regulation of three transporter genes associated with ZNF521 transduced cells more so than those transduced with MA9 alone, (SLC26A2, SLC16A9, and SLC38A5) (Figure 5C and Supplementary Table S1 (5)). The SLC38A5 transporter in particular promotes the uptake of nutrients for glutaminolysis and the serine-glycine-one-carbon pathway [55], which promotes the proliferation, survival, and growth of cancer cells.

### 2.5. Effect of ZNF521 Silencing on Growth of Human Leukemia Cells Expressing the MLL-AF9 Fusion Protein

**Figure 6:**
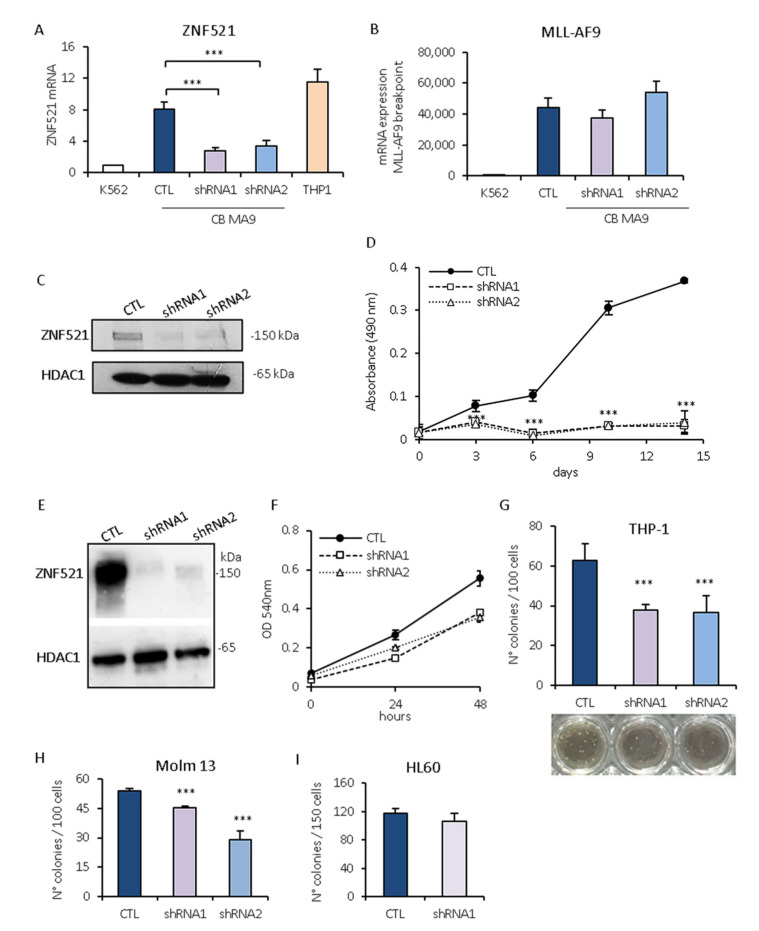
Effect of ZNF521 silencing in CD34^+^ hematopoietic stem/progenitor cells. CB-MA9 transduced cells cultivated for 30 days were silenced for ZNF521 with specific shRNA transduction, and the levels of ZNF521 (**A**) and the MLL-AF9 rearrangement (**B**) were analyzed by RT-Q-PCR. Expression was normalized by GAPDH mRNA expression, and the data represent the means ± SD of three replicates. (**C**) Immunoblotting analyses with nuclear extracts showed ZNF521 silencing in CB-MLL-AF9 cells; the signal was normalized to the nuclear protein HDAC1. (**D**) Cell viability was tested using the CellTiter 96^®^ Aqueous One Solution Assay: 1 × 10^3^ cells were plated in 96-well plates in complete medium, and absorbance at 490nm was measured after 2 h. Each point represents the mean ± SD of four replicates. (**I**) Effect of ZNF521 silencing in AML cell lines: THP-1 cells highly expressing both MLL and ZNF521 and were transduced with shRNAs for ZNF521 or with a non-silencing control shRNA. Protein lysates, which were prepared five days post-infection, were analyzed for ZNF521 expression by immunoblotting. (**F**) The proliferation of these cells was investigated by MTT assay; data are represented as mean ± SD. (**G**–**I**) Methylcellulose colony-formation assay was used to evaluate the effect of ZNF521 silencing on THP-1, MOLM-13, and HL60 cell lines. ZNF51 silencing resulted in a significant decrease in the number of colonies formed in cells expressing the MLL-AF9+ fusion protein, THP-1, and MOLM-13. The number of CFU did not change in HL60 cells negative for MLL rearrangements. The effects of ZNF521 silencing were compared to non-target shRNA control cells. Data are represented as mean ± SD (*** *p* < 0.001).

To consolidate the above-reported results, we silenced ZNF521 expression in the CB-MLL-AF9 transduced immortalized primary cells. Using two shRNA lentiviral vectors targeting ZNF521, the expression levels of ZNF521 were confirmed to be reduced by Q-RT-PCR and immunoblotting (Figure 6A,C), whereas MLL-AF9 was unaffected (Figure 6B). When ZNF521 was silenced in CB-MLL-AF9, cell viability showed a dramatic decrease (Figure 6D).

Additionally, we also used the THP-1 human leukemia cell line carrying an MLL-AF9 rearrangement and expressing high amounts of ZNF521. The silencing of ZNF521 (Figure 6E) induced a decrease in the growth rate compared to the control (Figure 6F) and was accompanied by a reduced clonogenic ability (about 50%) (Figure 6G). The results were confirmed in MOLM-13 cells, which also harbor an MLL-AF9 translocation, which were similarly affected by the silencing of ZNF521, as shown by reduced colony formation (Figure 6H); as a negative control, ZNF521-negative HL60 cells were used and displayed no significant change in colony formation upon ZNF521 knock-down (Figure 6I).

These data strengthen the functional cooperation between ZNF521 and MA9, resulting in the development, maintenance, and clonal expansion of leukemic cells.

## 3. Discussion

ZNF521 was initially discovered using a strategy to identify molecules that were selectively expressed in human hematopoietic stem and progenitor cells and that had a potential role in the regulation of the immature cell compartment [28,56,57,58,59,60]. In normal HSCs, the expression of ZNF521 rapidly diminishes, whereas in AML, and especially in those with MLL-rearrangements, expression remains elevated after oncogenic transformation [14,23,28,40,61,62]. ZNF521 undergoes transformation pre-transcriptionally as well as post-transcriptionally. It has been shown that ZNF521 is a transcriptional target of MLL-AF9 [26]; ZNF521-promoter occupancy by MLL-AF9 occurs together with a significant degree of H3K79me2 histone methylation, owing to the methylase activity of MLL-AF9 [25]. The hematopoietic transcription factors SPI1 (PU.1) and HOXC13 synergistically positively regulate the promoter for mZfp521 expression in B cells [63].

The expression of ZNF521/zfp521 can also be controlled by miRNAs in several different cancers, where miRNA expression is inversely correlated with that of ZNF521 [64]. Zfp521 is additionally found to be post-transcriptionally targeted for SUMOylation, facilitating erythroid hematopoiesis, and by SIAH2, a ubiquitin ligase for the degradation of ZFP521 in the adipocyte pathway [65,66].

ZNF521 functions as co-factor by transcriptionally activating or repressing a variety of genes.

An overexpression of ZNF521 interferes with the mechanisms that govern B-lymphoid [33] and erythroid differentiation [30] by inhibiting EBF1 and GATA1, respectively, thus resulting in a differentiation block; at the same time, the undifferentiated cells divide and undergo self-renewal. Differentiation delays have been documented in mesenchymal cells once ZNF521 is overexpressed [67,68,69,70] as well as in neural stem cells [71]. Moreover, ZNF521 has been implicated in the self-renewal of embryonic stem cells [72] as well as in HSCs [27] through interaction with HDACs via its N-terminal motif.

In this paper, we used data mining and gene expression analysis to dissect the profile of ZNF521 mRNA in different leukemias. ZNF521 expression was lower in FAB-AML-M3 patients and was higher in the M0 subgroup, both of which derive from immature hematopoietic cells, which is coherent with its proposed role in the immature HSC compartment. Notably, ZNF521 was found to be associated with specific cytogenetic profiles: (i) relatively low expression levels correlating to t(8;21), (ii) modest levels with t(15;17), (iii) high levels were found with the inv(16), and (iv) the highest amounts with t(11q23) and the other most common MLL rearrangements.

MECOM (EVI1), a gene overexpressed in AML, was induced by ZNF521 and is known to be induced by MLL rearrangement [39]. Since the EVI1 and PML-RARα myeloid oncoproteins have been described to act by aberrantly recruiting HDACs, leading to chromosomal remodelling [2], it is also likely that ZNF521 could contribute to this mechanism in an HDAC-dependent manner [51]. MLL-AF9 AML cells are sensitive to HDAC depletion and inhibitors, such that this epigenetic aspect can be considered as part of the cooperative mechanism of action [73].

In contrast, there was a distinct negative correlation with FLT3-ITD and NPM1 mutations, which involved different (to that of ZNF521/MLL-AF9) mechanisms of oncogenic transformation [74]. Other mutations in CEBPα and IDH2 were negatively or positively associated with ZNF521 although with reduced significance. CEBPα has two mutational hot spots, which result in reduced DNA binding or dimerization and contribute to a block in the transcription of granulocyte differentiation genes, resulting in maturation arrest [74]; its negative correlation with ZNF521 can only be found when both are mutated.

In this work, we have also investigated whether ZNF521 can functionally work with MLL-AF9 to promote the growth of transformed leukemic cells. The lentiviral transduction of MLL-AF9 was able to immortalize the CB-CD34^+^ stem cells such that the cultures grew significantly beyond 2 months. In addition, the co-transduction of MLL-AF9 and ZNF521 in CB-CD34^+^ cells further enhanced cell growth rates, resulting in an increase in the total cell population. Given the parallel increase the in number of colonies derived from these cultures, it can be predicted that the number of progenitor cells in the initial cultures increased. Such an increase in clonal expansion can be likely attributed to the promotion of the self-renewal of the HSCs owing to the combination of ZNF521 and MLL-AF9. It was found that MLL-AF9 acts to transform the HSCs to form LSCs, where the normal initiation of MLL transcriptional activity is aberrantly combined with the elongation activity of AF9 such that an uncontrolled proliferation develops [9]. When ZNF521 and MLL-AF9 act in combination for the promotion of self-renewal of both the HSC and LSC populations, this leads to a synergistic increase in the cellular expansion of “leukemic” cells. ZNF521 levels are crucial and affect the leukemic phenotype in AML, which is sustained by the MA9 fusion gene.

In agreement with the critical role played by the combination of ZNF521 and MLL-AF9, the shRNA-mediated silencing of ZNF521 leads to a rapid extinction of CD34^+^ MLL-AF9-immortalized cells. ZNF521 was also studied in THP-1, a cell line with the MLL-AF9^+^ translocation that is commonly employed as a model for AMLs, whose elevated ZNF521 expression levels makes it amenable to study the biological effects of ZNF521 knock-down. shRNA-mediated silencing of ZNF521 in THP-1 cells resulted in impaired growth and clonogenicity compared to cells transduced by the non-target shRNA vector.

The transcription profiling of the transformed cultures with ZNF521 and MA9 resulted in the induction of many of the known MA9 targets as well as others that can be predicted to contribute of the leukemic phenotype. The candidate genes we have analyzed here fall into different categories: transcription factors, epigenetic enzymes for chromatin remodeling proteins, cell cycle-associated genes, and transport or uptake proteins to sustain transformed cells with sufficient metabolites for the high proliferation that is typical in leukemic blasts.

These changes are evident at either the early stages of leukemia development or at a later time point, when transformed cells acquire their oncogenic phenotype. The genes up-regulated at early time points are likely derived from direct transcriptional epigenetic mechanisms that are associated with the MLL-AF9 gene, which, in turn, indirectly activate other transcription factors and pathways that lead to their transformation into leukemic blasts in the later stages of the process. Other transcriptional regulatory factors that are induced by both ZNF521 and MA9 at the early stages of leukemia development have various roles in controlling transcriptional activation, and some have been more recently associated with leukemias. Those with high levels of up-regulation, together with high hazard values and poor prognosis, should merit follow up studies to assess their roles in leukemogenesis.

## 4. Materials and Methods

### 4.1. Gene Expression Analysis from Public Repositories

The correlation between the expression levels of ZNF521 mRNA in AMLs and a series of parameters such as cytogenetics, FAB classification, and molecular profiling were investigated by analyzing publicly available data sets. The Microarray Innovations in LEukemia (MILE) study data set (GSE13204) of mixed leukemias [35] in the R2 database, the pediatric AML den Boer data (GSE17855) [36], and the pediatric AML data for MLL rearrangements (GDS4210, GSE19577) [40,61] were analyzed for ZNF521 gene expression in the different cytogenetic karyotypes. The Delwel data sets for AML comprising pediatric (GSE22056) and adult (GSE6891) patients [37] were analyzed for FAB classification and ZNF521 gene expression as well as for associations with FLT3-ITD, FLT3-TKD, CEBPα, NPM1, IDH1, IDH2, EVI1 (MECOM), KRAS, and NRAS mutations. Data were downloaded and analyzed by GraphPad PrismOne way analysis of variance (ANOVA), which was applied to assess global gene expression differences across considered groups.

### 4.2. Cell Culture

Cord blood (CB)-CD34^+^ cells were purchased from Lonza and were cultured as 0.5–1.0 × 10^5^ cells/mL in Xeno-free StemMACS™ HSC Expansion Media (Miltenyi Biotec Bologna, Italy) in the presence of Thrombopoietin (TPO), FLT3-Ligand (FLT3-L), and Stem Cell Factor (SCF) (100 ng/mL). These primitive hematopoietic cells were characterized by flow cytometry (BD FAC-S-can II) and comprised 99.8% CD34^+^ cells. Lentiviral-transformed cells were cultured in MyeloCult™ H5100 (STEMCELL Technologies, Milan, Italy) supplemented with 1 μM of hydrocortisone (HC) and 10 ng/mL of SCF, FLT3-L, and Interleukin 3 (IL3).

THP1, MOLM-13, and HL60 cells were cultured in RPMI-1640 medium. HEK293T were cultured in Dulbecco’s modified Eagle medium (DMEM). Media were supplemented with 10% fetal bovine serum (FBS), 50U of penicillin, and 50μg/mL of streptomycin. Cells were maintained at 37 °C in a 5% CO2 humidified incubator.

### 4.3. Cell Transduction

Lentiviral particles were packaged by the co-transfection of 10 μg of the pCMV Δ8.9 and 2 μg of the VSVG vectors (vesicular stomatitis virus glycoprotein), encoding for the packaging genes and envelope, respectively, together with 10 μg of transfer vector plasmids (UMG-LV6-ZNF521 or UMG-LV6-MA9 alone or in combination) [75] or 5 μg of each ZNF521-shRNA were used (Sigma-Aldrich, Milan, Italy). Two lentiviral vectors were used for ZNF521 silencing: FG12-H11 (shRNA-1) (GCUAAAGAUAGUAAUGCATT) and FG12-H12 (shRNA-2) (UGUGGAACAAAUGGAGCUUTT), which have been previously described to inhibit ZNF521 expression. As a control, the human non-target mission lentiviral vector was used according to the manufacturer’s instructions (Sigma-Aldrich Milan, Italy).

HEK293T 2 × 10^7^ cells were transfected using a standard calcium phosphate transfection protocol. After a 6 h incubation period at 37 °C, the medium was changed, and IMDM 3% FBS was added. The supernatant containing the viral particles were collected after 24 and 48 h, filtered (0.45 μm cellulose acetate), and added at an MOI of 2 to 2.5 × 10^5^ CB-CD34^+^ cells. The cells were then subjected to two consecutive cycles of spin inoculation (700× *g* at 32 °C for 40 min) in the presence of 8 μg/mL of polybrene (Sigma-Aldrich, Milan, Italy). For cell surface staining, cells were labeled with fluorescent-conjugated antibodies for CD34 (Miltenyi Biotec, Bologna, Italy) in the dark at 4 °C for 30 min and were then washed with cold PBS. Cells were re-suspended in 300 μL of PBS and were acquired on the BD FACscan™ II. Data analysis was performed using FlowJo software 8.8.6 (Becton Dickinson, Milan, Italy); the CD34^+^ cells were gated in the right upper panel.

### 4.4. RNA Isolation, Reverse Transcription, and Quantitative RT-PCR

Total RNA was extracted with the TRI Reagent (Sigma-Aldrich, Milan, Italy). cDNA was synthesized from 1 µg RNA using SuperScript III reverse transcriptase at 42 °C and 2.5 µM random hexamers (Life Technologies, Monza, Italy). Quantitative RT-PCR (Q-RT-PCR) was run using the SYBR™ Green master mix (Bio-Rad, Milan, Italy) and was analyzed using an iQ5 multicolor detection system (Bio-Rad, Milan, Italy). One cycle of 3 min at 95 °C was followed by 45 cycles of 10 s at 95 °C, 10 s at 60 °C, and 20 s at 72 °C followed by a melting curve. The results are expressed as mRNA levels normalized to the GAPDH mRNA expression in each sample [76]. To determine the mRNA levels, at least three independent RNA samples were analyzed.

The primer sequences used for the Q-RT-PCR were:

GAPDH, FWD: CACCATCTTCCAGGAGCGAG and REV: TCACGCCACAGTTTCCCGGA;

ZNF521, FWD: CCACATCCAAACCATCCACCG and REV: CAGGTGGCACTGGAGTTTGGC; MLL-AF9 break-point FWD: CACCTACTACAGGACCGCCAA and REV: CTAGGTATGCCTTGTCACATTCACC;

HOXA10 FWD: CTTCCGAGAGCAGCAAAGCCTC and REV: TCCAGTGTCTGGTGCTTCGTGT;

HOXA9 FWD: CCCCATCGATCCCAATAACC and REV: TCACTCGTCTTTTGCTCGGT

MEIS1 FWD: AAGCAGTTGGCACAAGACACG and REV: TGTCCATCAGGATTATAAGGTGTTCC;

MEF2C FWD: TCCACCAGGCAGCAAGAATACG and REV: GGAGTTGCTACGGAAACCACTG;

PBX3 FWD: CCAGTGAAGAAGCCAAAGAGGAG and REV: CAGCATAGAGGTTGGCTTCTTCC.

### 4.5. Continuous Culture Assays

CD34^+^ cells were plated at the initial seeding density of 2 × 10^4^ cells/48 well and then, different time points during the experiment, were re-plated at increasing amounts [77]. The cell counts and cellular dilution factors were recorded and were used to calculate the cumulative cell numbers and total progenitors.

### 4.6. MTT Assay

THP-1 cells (1 × 10^3^/0.1 mL) were plated in 96-well flat bottom plates and at time 0, 24 h, and 48 h, 10 μL of 5 mg/mL MTT solution was added to each well for 90 min. After incubation at 37 °C, an equal volume of stop solution (0.08 N HCl in isopropanol) was added to each well to dissolve the formazan crystals [78]. The absorbance at 540 nm was determined using an iMarkTM microplate reader (Bio-Rad Instrument, Milan, Italy). The experiments were performed in quadruplicate, and the results are shown as mean ± SD. * *p* < 0.05.

### 4.7. MTS Assay

CB-CD34^+^ cells (MLL-AF9) were lentivirally transformed and were then silenced for ZNF521 and assayed for proliferation using the CellTiter 96 AQueous One Solution Cell Proliferation Assay (MTS) kit (Promega, Milan, Italy) according to the manufacturer’s instructions. In brief, 1 × 10^3^ cells/96-well were plated and cultured for two weeks; cell viability was monitored at various time points: 20 µL/well of reagent was added and after 2 h at 37 °C, and the absorbance at 490 nm was recorded using the GloMax EXPLORER (Promega, Milan, Italy). The experiments were performed in quadruplicate, and the results are shown as mean ± SD * *p* < 0.05.

### 4.8. Colony Formation Assay

For colony formation assays, 100 or 150 transformed cells were plated in MethoCult media H4100 (StemCell Technologies, Milan, Italy) consisting of RPMI-1640 with 1% methylcellulose and 10% FBS. Primary transformed cell colony assays were performed with the StemMACS HSC-CFU complete with Epo (Miltenyi Biotec, Bologna, Italy). In brief, 2 × 10^2^ cells/mL or increasing amounts (of cells) (58 days) were added to methylcellulose at different time points during the experiment and were gently vortexed to suspend the cells evenly. The cells were plated in 4-well plates (500 μL methylcellulose/well) using an 18-gauge blunt needle and were maintained at 37 °C, 5% CO_2_ for 14 days. Colonies were then observed through an inverted microscope and were scored. Assays were performed in triplicate.

### 4.9. Protein Lysates and Immunoblotting

To obtain nuclear extracts, cells were lysed in hypotonic buffer containing 10 mM Hepes pH 7.9, 10 mM KCl, 0.1 mM EDTA pH 8.0, protease inhibitors (P8849, Sigma, Milan, Italy), and phosphatase inhibitor cocktails 2 and 3 (P0044, P5726 Sigma) and were incubated on ice for 20 min. Igepal-630 (NP40) 0.25% was added to the cell lysates, and samples were centrifuged for 15 min at 14.000× *g*. The pelleted nuclei were resuspended in a hypertonic buffer containing 20 mM Hepes pH 7.9, 0.4 M NaCl, 1 mM EDTA pH 8.0, and protease and phosphatase inhibitors. The nuclei were subjected to three rounds of alternating vortex mixing and ice-cooling, were centrifuged at 15.000× *g* for 20 min, and the supernatants containing the nuclear extracts were recovered. The Bradford assay was used to measure the concentration of the protein extracts.

Protein extracts (30 µg) were electrophoresed on 4–12% NuPAGE Novex bis-Tris gradient polyacrylamide gels (Life Technologies, Monza, Italy) under non reducing conditions and were electroblotted onto nitrocellulose. Membranes were blocked for 30 min with Blotto Milk 5% in PBS–0.05%Tween20, incubated with anti-ZNF521 (S-15 EHZF—Santa Cruz Biotechnology, Milan, Italy) (1:1000) and anti-HDAC-1 (H3284—Sigma-Aldrich, Milan, Italy) (1:12,000) at 4 °C overnight, rinsed, and then incubated for 1 h with anti-rabbit secondary HRP-conjugated antibody (Santa Cruz, Biotechnology, Milan, Italy) and visualized with the ImmunoCruz Western blotting luminal reagent (Santa Cruz, Biotechnology, Milan, Italy) by means of autoradiography.

### 4.10. Global Transcriptome Profiling

Total RNA was purified from duplicate samples by resuspending in TRI Reagent (Sigma-Aldrich) and freezing it at −80 °C before processing. Uniformly transduced proliferating cultures (over 95% GFP^+^) at 23 days were compared to the control (CTL) empty vector (UMG-LV6-GFP), which ceased to grow at later time points. These were compared to the later time point of 58 days, when the double transduction of UMG-LV6-MA9 plus UMG-LV6-ZNF521 practically immortalized the cells, representing a leukemic phenotype model.

Total RNA samples were processed using WT PLUS Reagent Kit according to the manufacturer’s protocols (Thermo Fisher Scientific, Waltham, MA, USA). Wide mRNA-transcriptome profiling was assessed using Clariom D Human array (Thermo Fisher Scientific, Waltham, MA, USA), as previously reported [79].

Robust Multichip Average (RMA) processing was applied on raw data based on the platform design (pd.clariom.d.human), allowing background subtraction, quantile normalization, and median-polish summarization on the core probe sets by using the oligo package in an R/Bioconductor environment (R version 3.5.1) [80].

Global expression levels of 138.745 total transcripts were extracted from the Expression Set. Further analyses were performed on 20.558 total annotated features after filtering the transcripts on the base of the Entrez Gene Identifier and after the exclusion of non-coding or uncharacterized protein transcripts, as previously reported [81].

Hierarchical clustering analysis was performed on the top 1% of transcripts with higher variance across the entire dataset. The expression data of the scaled and filtered transcripts were analyzed by applying the Euclidean distance measure, using the dist function in R, and using the average linkage method by means of hclust function in R.

Differential expression analysis was performed on logged2 transformed data by means of RankProduct [82], a non-parametric method for identifying differentially expressed (up- or down-regulated) genes based on the estimated percentage of false predictions (*pfp*).

Heatmap.2 function from the gplots package was used to represent transcript lists.

Gene set enrichment analysis (GSEA, version 4.1.0) [83,84] was performed under default analysis conditions on the Hallmark and KEGG gene set collections (Molecular Signatures Database v7.4) by applying 1000 gene set permutations. Significant gene sets were chosen on the base of the nominal *p*-value < 0.05 and the FDR *q*-value < 25%.

Functional annotation clustering analysis was performed on the gene ontology (GO) terms by means of The Database for Annotation, Visualization and Integrated Discovery (DAVID) v6.8, available at https://david.ncifcrf.gov/ (accessed on 28 July 2021) under high stringency conditions [42]. Gene expression data were deposited under the GEO accession number GSE181006.

### 4.11. Survival Analysis

Survival hazard values for up- or down-regulated DEGs were derived from the Gepia data base (http://gepia.cancer-pku.cn/ (accessed on 28 July 2021) [56] for 178 patients with AML leukemias (LAML) for overall survival, where the hazards ratio was calculated from a medium value of a 50% high and 50% low division based on a Cox PH Model with a 95% confidence interval.

### 4.12. Statistical Analysis

Student’s *t* test was used to calculate all *p* values. Asterisks in the figures indicate differences with statistical significance: * *p* < 0.05, ** *p* < 0.01, and *** *p* < 0.001.

## 5. Conclusions

The majority of AML patients who are positive for MLL-AF9 also carry high ZNF521 levels and can be predicted to have a more robust leukemic phenotype with poor prognosis. By performing gene expression profiling in HSCs in which the expression of both MLL-AF9 and ZNF521 was modulated by lentiviruses, we extended the panel of the genes and pathways potentially involved in this leukemia, which will help to design better targeted therapies. Taken together, our data strengthen the role of ZNF521 in the regulation of the immature compartment of malignant hematopoiesis, which, by altering the gene expression landscape, contributes to the development and/or maintenance of AML acting in concert with the MLL-AF9 fusion oncogene.

## Data Availability

GSE181006 study: https://www.ncbi.nlm.nih.gov/geo/query/acc.cgi?acc=GSE181006 (accessed on 28 July 2021).

## Supplementary Materials

The following are available online at https://www.mdpi.com/article/10.3390/ijms221910814/s1.
